# Preventable fatal injury during rally race: a multidisciplinary approach

**DOI:** 10.1007/s00414-020-02470-2

**Published:** 2020-11-25

**Authors:** Stefania Zerbo, Clio Bilotta, Giulio Perrone, Ginevra Malta, Giuseppe Lo Re, Maria Chiara Terranova, Antonina Argo, Sergio Salerno

**Affiliations:** 1 Department of Health Promotion, Mother and Child Care, Internal Medicine and Medical Specialties, Section of Legal Medicine, University of Palermo, Via del Vespro 129, 90127 Palermo, Italy; 2 Radiology Section, DIBIMED, University of Palermo, Via del Vespro 129, 90127 Palermo, Italy

**Keywords:** Rally race, Injury, HANS collar, Forensic pathology, Postmortem CT

## Abstract

**Introduction:**

The motor vehicle crash (MVC) constitutes an important challenge for forensic pathology in order to identify the manner and cause of death. Our study focuses on a fatal accident during a rally race corresponding to MVC sub-category.

**Materials and method:**

Postmortem computed tomography (PMCT) was performed before the conventional autopsy. Autoptic and PMCT data were compared. Data collection allowed analyzing biomechanical dynamics of the incident and post-traumatic injuries through qualitative-statistics and solicitation quantitative indices.

**Results:**

Photo and circumstantial evidence analysis showed a wrong installation of double shoulder belt system of head and neck support (HANS) collar. PMTC clearly highlighted multiple and bilateral fractures involving roof and base of skull; a displaced fracture of the right acetabulum was also encountered. Autopsy confirmed PMCT data and revealed a brainstem laceration. AIS (Abbreviated Injury Scale) achieved a maximum score in consideration of fatal injuries.

**Discussion:**

The injuries analysis resulting from photographic surveys examination, conventional autopsy, and PMCT has led us to confirm a fatal front collision with a tree trunk. Head trauma represents a major injury in the present case. In this case, head injuries, related to whiplash trauma, are a consequence of a double shoulder belt system (HANS collar component) wrong installation.

**Conclusion:**

MVC and especially high-speed motor racing represent an important death cause. There was, for this reason, a marked development of cars and occupants’ safety systems, such as HANS collar. PMCT improves the diagnostic performance of conventional autopsy and increases forensic medical knowledge related to traumatic injuries.

## Introduction

The motor vehicle crash (MVC) constitutes an important challenge for forensic pathology, especially in recent years. Our study focuses on a fatal accident during a rally race; therefore, it corresponds to MVC sub-category.

Rally is a motorsport discipline taking place on public, asphalt or dirt, road. Modern rally competitions are developed since the beginning of the twentieth century in Europe. The “Mille Miglia” race, the most prestigious and ancient race, can be considered an ancestor of this motorsport discipline. The concept of the rally was vague, and there are no official regulations until the first half of the 1960s. Rally races became competitions with official regulations around the first half of the 1970s. During a rally race, pilots may drive only a series of cars. Rally competition divides into two types of stages: special stages and transport stages. The last one consists of a route marked out on Radar or Road Book that must be performed within a specific time limit. In these stages, penalties are applied for being completed either too fast or too slowly. Special stages are tests of skill clockwork, in which pilots drive on winding and rough roads, in the absence of road safety equipment. Rally races can be considered regularity races during transport stages, in which pilots must comply with road traffic and timelines regulations, and time trial during special stages.

**Fig. 1:**
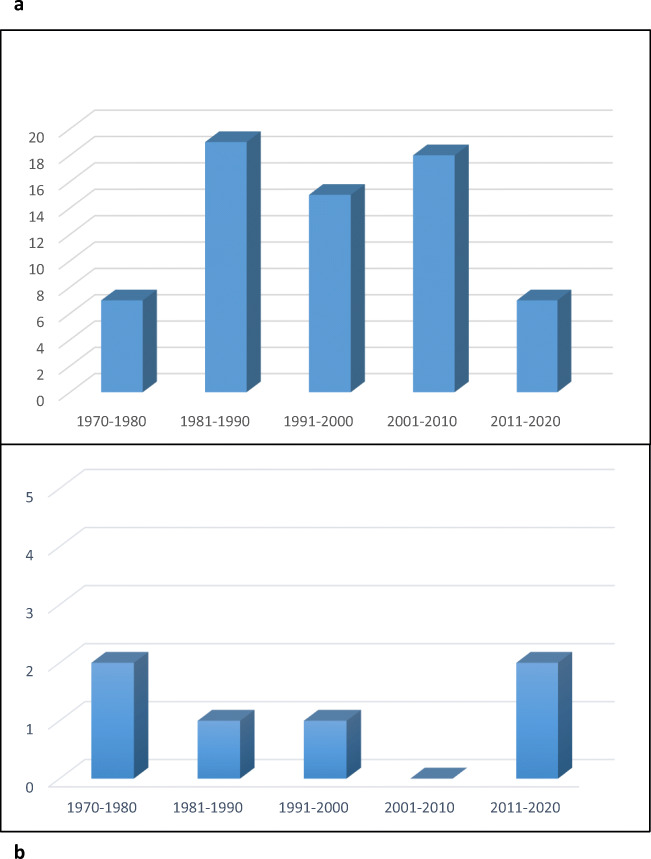
**a** Death number (*n*) in rally races from 1970 to 2020; **b** death number (*n*) in Italian Rally Championship from 1970 to 2020

Epidemiological data (Table 1, Fig. 1) show an increase in fatal MVC from the 1980s. It could be a consequence of the increasing speed of rally cars. The marked progress in polytraumatized patients’ therapy, in the medical field, and occupant’s safety system, in the engineering field, on the other hand, decrease the rally mortality rate in the last decade. The safety cycle, a security system for road accidents, is, in fact, fundamental for the development of a specific prevention system. It includes several surveillance mechanisms, biomechanical studies carried out through crash-tests, and analysis of epidemiological data on vehicles, drivers, and places in the MVC.

**Table 1:** Death number (*n*) in continental and world Rally Championships from 1970 to 2020

	1970–1980	1981–1990	1991–2000	2001–2010	2011–2020
European National Series and National Rallies	1	2	6	4	3
Eaupean Rally Championship	0	2	3	6	0
African Rally Chapionship and African Rallies	1	1	0	3	0
Ocean National Series and National Rallies	1	2	4	4	3
SCCA ProRally and Rally America	2	2	1	0	1
World Rally Championship	2	10	1	1	0
Total	7	19	15	18	7

In the illustrated case, a car accident occurred during “Targa Florio” rally, an ancient Sicilian car racing competition that usually takes place in the mountain range of Palermo province in May. Vincenzo Florio, a citizen of Palermo’s wealthy family, created, financed, and organized “Targa Florio” race. It was raced 61 times, with no solution of continuity, from 1906 to 1977. It was turned into a rally race for safety reasons in 1978, remaining one of Italian and European Rally Championship stages. The highest mortality rate was recorded in the decade 1970–1980 in “Targa Florio” rally race (Table 2).

**Table 2:** Fatal accidents during Targa Florio from 1920 to 2020

Time	Death number		
	Pilot	Referee	Spectators
1926	1		
1934	1		
1958	1		
1971	1		
1973	1		
1977			2
1985	1		
1991			1
2012	1		
2017	1	1	

In the case of MVC, it is necessary to check whether injuries, resulting from impact, are enough to cause death, or it is a necessary but not sufficient condition. In this last case, the pre-existing diseases could induce an abnormal response to trauma. It is, therefore, important to exclude that MVC is a consequence of an acute pathological event, prior to the incident. Autopsy remains one of the main data sources for fatal crashes; it is fundamental to answer car accident questions regarding the kind and cause of death.

Autopsy has recently been integrated with a radiological investigation, which is necessary to accurately define injuries before the autopsy and to guide medical examiner during the one. There has been an increased use of Postmortem Computed Tomography (PMCT) in the forensic field recently. The postmortem examination remains, however, the gold standard. PMCT allows the dynamic reconstruction of the MVC in a noninvasive way. Three-dimensional volume rendering (3DVR) imaging in radiological applications allows to obtain three-dimensional reconstructions of the whole body [1]. Medico-legal and postmortem radiological synergic investigates injuries more thoroughly, improving prevention and safety system. PMCT advantages, such as short execution time or objective and reproducible medical records, full depiction of fractures and lesions hardly detectable at the autoptic examination, make it ideal for synergy and integration with medico-legal investigation. [2, 3]

## Case report

The illustrated case concerns a fatal accident that occurred during a rally race. The pilot was driving his rally car on a straight road after a curve when he lost control of the vehicle, maybe because of wet asphalt. The vehicle went off the road, running over a referee, and crashing into a tree. The pilot and referee have died on impact; the copilot survived.

The pilot’s body was transferred to a University Hospital Morgue, and it underwent a medico-legal examination. The investigation was supplemented by a preliminary PMCT 18 h after death. All radiologic scans were performed by two forensic experienced board-certified radiologists.

Photographic survey of places, collected by judicial police and videos of first help, have been provided to analyze it and better understand the dynamics of the incident.

### PMCT

The postmortem interval between PMCT and the medico-legal autopsy was about 6 h. We performed a non-contrast whole/body scan of the victim enveloped in a bag before a conventional autopsy. PMCT was performed with a 128 slices MDCT scanner (Somatom Definition AS®, Siemens Healthcare Erlangen Germany) using:

- Tube voltage of 120 kVp, with an effective tube current of 120–160 effective mAs;
- Gantry rotation time of 0.5 s, beam pitch of 1.2, and table speed of 46 mm per gantry rotation;
- Overlapped slices with a thickness of 0.6 mm [espr guidelines].

Images were reviewed using our institutional PACS viewer (Elephant.net suite® AGFA Health Care N.V., Belgium) and dedicated workstations (Singovia® Siemens Healthcare Erlangen Germany; Horos Project, Pixmeo). Bone imaging algorithms, soft-tissue algorithms for the whole-body examination, dedicated head and lung algorithms for the brain, and pulmonary evaluation respectively were used. Due to the impossibility of acquiring the whole body in a single scan, five scans of the body (the head and body trunk, the arms, and the legs) were repeated after repositioning. Images are then evaluated using multiplanar reformatting (MPR) in coronal and sagittal planes and volume-rendering (VR) elaboration with a dedicated bone lung program.

### Autopsy and biomechanical injuries analysis

Autopsy is based on the collection and analysis of postmortem data. We took different organs’ samples after the autopsy. The samples were stored in 10% neutral buffered formalin. Hematoxylin-eosin-stained tissue sections were analyzed with an optic microscope with 4-10-40-100 zoom. We carried out an analysis of drugs and toxic substances in biological fluids.

The assessment of post-traumatic injuries found through PMCT and autopsy is based on the evaluation of several quantitative and qualitative indices. Qualitative-statistics (AIS, MAIS, IIS) and solicitation quantitative (HIC, NIC, TBI) indices and their respective tolerance threshold may be taken into consideration in the medico-legal field [4, 5], not only for MCV-related deaths but also for other traumatic deaths, such as those caused by work-related accidents, sporting accidents, explosions, mass disasters [6]. The Abbreviated Injury Scale (AIS) is an anatomical evaluation system, based on the classification of each injury depending on their severity and location, through a scale from one to six (Table 3, 4). The number “one” corresponds to mild lesions, the number “six” to fatal injuries. The injuries can be placed on nine different anatomical areas regarding anatomical criteria. AIS [7] represents the life-threatening secondary to each injury; it does not provide a complete indication of the overall clinical picture of the patient.

**Table 3:** AIS scale

AIS value	Injury description
0	No injury
1	Minor
2	Moderate
3	Serious
4	Severe
5	Critical
6	Maximum/fatal

**Table 4:** AIS injuries classification

AIS	Severity	Injury description	Body region included
1	Minor	Sprains, mild concussions, abrasions.	Upper and lower extremities, face
2	Moderate	Dislocations, lacerations, concussions, mild fractures	Upper and lower extremities, face, head
3	Serious	Fractures, ruptures, serious concussions, cerebral concussions	Upper and lower extremities, face, head, abdomen
4	Severe	Serious lacerations, serious contusions, multiple fractures.	Thorax, brain
5	Critical	Various	Brain
6	Unsurvivable	Injuries ultimately result in death	Thorax, spinal column

We have classified the injuries, depending on severity, location, and involved biological tissue (whole body surface, nerves, vessels, bones).

Finally, we compared injuries found at PMCT and autopsy.

## Results

### Findings of analysis of photographic survey and circumstantial evidence

Photo and circumstantial evidence analysis showed a wrong installation of a double shoulder belt system of the head and neck support (HANS) collar, an important safety device for a helmet. The pilot did not properly wear HANS-belts upon HANS-yoke and did not cross them. He wore body belts correctly, as confirmed by engineering expertise.

### PMCT findings

**Fig 2:**
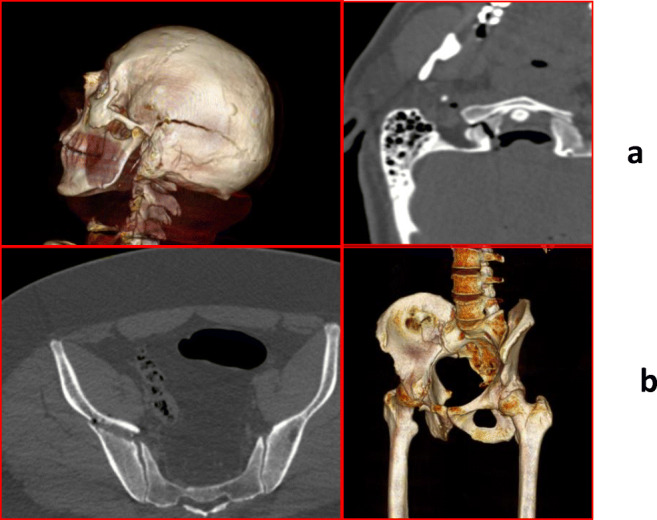
**a** Axial image and 3DVR reconstruction image of head clearly shows the mastoid and basic of skull fracture, extended to the left parietal bone; **b** axial image and 3DVR reconstruction image of head clearly right-side displaced acetabulum

PMCT clearly showed a huge mastoid and basic of skull fracture (Fig. 2a), mainly in the right side of the basis of skull and extended to the left parietal bone, fracture of the right side of the atlas. Signs of pneumocephalus, ventricular hemorrhages, and small subarachnoid hemorrhages were also highlighted. We also found an acetabular fracture. A displaced fracture of the right acetabulum (Fig. 2b) was also encountered.

### Autopsy findings

**Fig 3:**
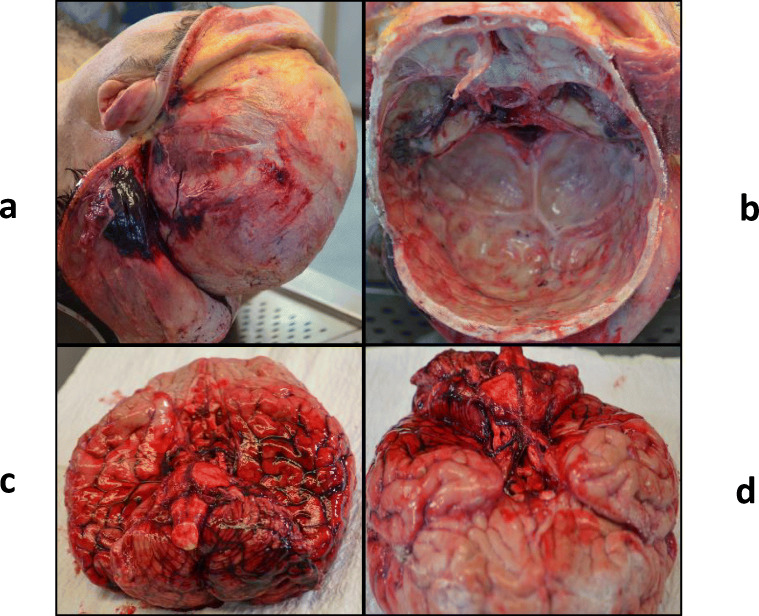
**a** Hemorrhagic infiltration of the scalp inner surface and galea capitis. **b** Basicranial fractures. **c** Subarachnoid hemorrhage in left temporal-parietal portion of the brain and cerebellum. **d** brainstem laceration

The body was 178 cm in length, weighing 90 Kg. At the external examination, bilateral otorrhagia, abrasions, and bruises on the right side of the lateral cervical region and acromioclavicular area were found. Skull section showed hemorrhagic infiltration of the inner scalp surface, galea capitis, periosteum in the left temporal-parietal-occipital portion of the skull; a skull fracture extended from the temporal bone to the occipital bone, involving also left parietal bone (Fig. 3a, 3b). Subarachnoid hemorrhage in the left temporal-parietal portion of the brain and cerebellum, leptomeningeal congestion (Fig. 3c), and a laceration in the forepart of the brain stem (Fig. 3d), involving both cerebral peduncles, were highlighted. Basicranial fractures as a line with horizontal extension in the middle cranial fossa, also involving sella turcica, great wings of the sphenoid, bilaterally, and squamous part of the temporal bone, were also found; a fracture with horizontal extension in the left posterior cranial fossa, involving the squamous part of occipital bone; a fracture line in the right side of the foramen magnum (ring fractures). Sternoclavicular dislocation, pulmonary emphysema, contusions, and perirenal bleeding were highlighted through thorax and abdomen examination. Pelvis section confirmed acetabular fracture. Autopsy did not reveal other concomitant acute pathological events.

### Histological and toxicological findings

**Fig. 4:**
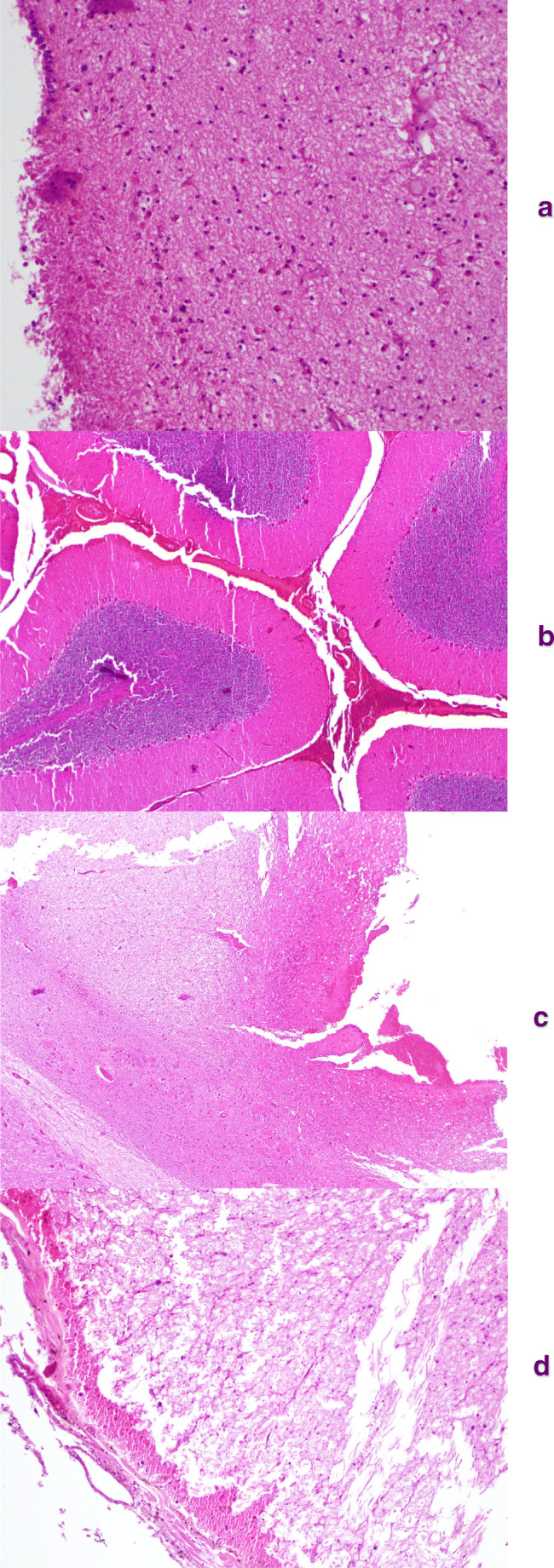
**a** Brain (× 10 zoom): neuronal degeneration and parenchymal necrosis; **b** cerebellum (× 4 zoom): hemorrhage in the cerebellar membranes; **c** brainstem (× 4 zoom): neuronal degeneration, edema, and subpial and intraparenchymal hemorrhage; **d** medulla oblongata ((× 10 zoom): neuronal degeneration, edema, and subpial and intraparenchymal hemorrhage

The histological slides reading showed parenchymal necrosis in the right parietal lobe, vascular congestion in the occipital lobe, perivascular edema in the cerebral membranes, hemorrhage in the cerebellar membranes, neuronal degeneration, edema, and subpial and intraparenchymal hemorrhage in the brain stem were highlighted (Fig. 4). Non-obstructive sclerosis of the left coronary artery was found. Focal bronchoalveolar hemorrhage and atelectasis, widespread emphysema was also highlighted. Perirenal hemorrhage was confirmed.

Neither drugs nor ethanol was detected by screening toxicological analysis.

### Injuries classification and analysis

Injuries analysis (Table 5) showed five types of head injuries: cranic fractures with AIS value equal to 3; subarachnoid hemorrhage with AIS value equal to 6; cerebrum pneumocephalus with AIS value equal to 4; cerebellar hemorrhage with AIS value equal to 6; and brainstem laceration with AIS value equal to 6. One type of vertebral injury: C1 fracture with AIS value equal to 6. Two types of thoracic injuries: pulmonary emphysema and contusions with AIS value equal to 4; sternoclavicular dislocation with AIS value equal to 2. One type of abdominal injury: perirenal bleeding with AIS value equal to 3.

**Table 5:** Injuries classification legend: X, positive response; 0, negative response

Injury	AIS	Detection	
		Autopsy	TC
Cranic fractures	3	X	X
Subarachnoid hemorrhage	6	X	X
Cerebrum pneumocephalus	4	0	X
Cerebellar Hemorrhage	6	X	X
Brainstem lacerations	6	X	0
C1 fracture	6	X	X
Pulmonary emphysema and contusions	4	X	X
Sternoclavicular dislocation	2	X	X
Perirenal bleeding	3	X	X

All injuries were found through both diagnostic methods (PMCT, autopsy), except cerebrum pneumocephalus, clearly identified during PMCT and brainstem lacerations better appreciable at autopsy.

## Discussion

The injuries analysis during an autopsy, supplemented with PMCT and photographic surveys examination, has allowed us to carry a biomechanical analysis of the incident. The important damage to the front of the car (bumper and right-front tire), observed through photographic survey’s examination, has led us to confirm a front collision with a tree trunk. We also assume a high kinetic energy impact because of the considerable car damage. Front collisions are extremely dangerous [8] and represent 50-55% of major or fatal MVC.

The vehicle occupants may be exposed to considerably high stresses (accelerations or decelerations), caused by the impact, during car crashes; they may crash into vehicle interior structures because of inertial motion, and their body segments may be decelerated. The magnitude of the deceleration vector reaches the peak in milliseconds, and then it decreases to around zero.

Head trauma, with basicranial fractures and brainstem laceration, represents the major injury in the present case. Skull base involvement is an expression of high kinetic energy. Head directed flexion is one of the mechanisms of ring fractures at the skull base in occipital bone traumas; shearing effect occurs in facial and occipital bone traumas.

The support system for the head is composed of cervical vertebrae, acting as a head pivot, and neck muscle-tendinous structures, connecting the head to the pivot. Cervical vertebrae constitute the focus where the reaction force, opposed to damaging force, originates.

Damaging forces deform the skull massively, shortening the diameter between the point of damaging force application and vertebral column portion, in which reaction force originates. Skull fractures may occur following the violent head hyperextension or hyperflexion. Ring fractures at the skull base are the most common skull fractures in case of high energy MVC [9, 10]; they can be complete or incomplete. The incomplete ring fractures extend along the middle cranial fossa and behind the petrous pyramid of the temporal bone, bilaterally [11].

The inertial force, related to the acceleration due to blunt head trauma, leads a brain shift in the braincase, resulting in intraparenchymal contusions. Compressive tension and pulling forces can cause potential injuries. Combined dynamic forces come into play in head trauma more frequently; these forces are capable of producing a brain shift through two components: translation (linear motion) and rotation (angular acceleration).

Traumatic brain deceleration forces could cause a diffuse axonal injury (DAI) [12], characterized by alteration of axonal cytoskeleton, axonal transport disruption, and axonal microtubules misalignment. These changes induce a β-amyloid precursor protein (β-APP) accumulation in damaged axons in a time-dependent manner. Immunohistochemical techniques for β-APP are, in fact, performed to investigate DAI, showing high sensitivity for traumatic axonal injuries and providing additional information about survival time and degree of mechanical forces [13].

In this case, the brainstem, one of the vital organs, has been injured; there is a clear evidence of a pontomedullary laceration. A brainstem laceration induces an interruption of nerve conduction in the central nervous system, with immediate cardiopulmonary arrest and instant death. Cervical Whiplash trauma is able to produce brainstem and cervical lacerations [14], secondary to head and neck hyperextension-hyperflexion, due to a sudden acceleration-deceleration force. It can also cause neck injuries, such as ligaments and joint capsules lacerations, physiological lordosis alterations, and nerve damages. The drivers may have hinge fracture of cranial base if their head makes lateral movements during whiplash, ring fracture is a basicranial injury, with a fracture line running from side to side across the middle cranial cavities, separating the base into two halves, anterior and posterior. [15]

Head hyperextension causes injury of cervical spinal anterior longitudinal ligament and front of neck soft-tissues; the abrupt head flexion damages the back of the neck ligaments and muscles, such as sternocleidomastoid or scalenus muscle.

In this case, head injuries, related to whiplash, are a consequence of a double shoulder belt system (HANS Collar component) wrong installation. The pilot did not properly wear HANS-belts upon HANS-yoke and did not cross them as confirmed by engineering expertise. He wore body belts correctly.

To address this issue, it is necessary to premise that the double shoulder belt system allows a decrease in impact force and trunk movements. Head and neck pilots, therefore, are very vulnerable in case of impact, especially when the crash includes sudden movements on the frontal and transverse plane (anterior-posterior translation and flexion-extension movement). HANS collar was progressively introduced for pilot safety. In fact, it serves as a head and neck support; it allows to resist flexion, distraction, and deceleration movements, diverting translation head movement toward trunk [16, 17].

**Fig. 5:**
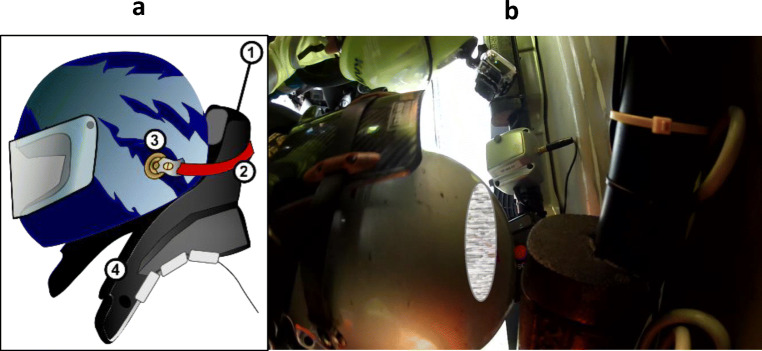
**a** HANS device components: 1—HANS collar, 2—tether, 3—helmet anchor, 4—shoulder support. **b** HANS device in the present case

The device is made up of different components (Fig. 5), each one with a different task:

- Safety-belt attached to the helmet: it links helmet to the device, allowing to a transmission of forces from head to the device during an impact;
- Helmet anchor: it needs to secure safety-belt to helmet allowing loads transmission through HANS collar;
- HANS collar: it transmits loads from anchor system to HANS-yoke;
- Safety belts anchoring system to the collar: it connects safety-belts with collar, consenting loads transmission from head to shoulders through the yoke;
- Yoke: it is situated on shoulder and thorax of the pilot, for load transmission to the trunk;
- The interface system with safety belts: it is the upper part of the yoke. It interfaces with safety belts, and it transfers forces from the head to trunk. [18]

Literary studies have highlighted that the use of this device significantly decreases tension and shear neck force, injuries secondary to flexion and distraction movements, and, therefore, head-spine traumas. HANS support device ensures greater pilot safety in car racing, decreasing in the sequels, secondary to MVC [19]. Several studies have shown that Hans collar allows a decrease in head motions and damaging force acting on the neck during frontal crashes, reducing pilot basicranial fractures and head impact on vehicle interior rigid structures [20, 21]. In conclusion, HANS device allows transmitting forces from head-neck to trunk [22].

## Conclusions

MVC and especially high-speed motor racing’s injuries represent an important death cause. There was, for this reason, a marked development of cars and occupants’ safety systems, such as HANS collar. It restricts head and neck movements, allowing a decrease in traumatic craniocerebral injuries. PMCT examination is really useful in the depiction of cranial fractures, allows a full depiction of fractures of the basis of the skull and hemorrhagic lesions, otherwise hardly detectable at the autoptic examination. The use of radiological diagnosis helps in the depiction of lesions, fasting the autoptic one, and improving the comprehension of death causes. Autopsy remains the gold standard, allowing analyzing injuries and excluding other death causes, so it would be totally wrong to claim that PMCT can replace autopsy. The combination of both diagnostic methods, however, is an advantage, especially in the case of multiple traumas secondary to an incident.
